# Malaria and Its Mode of Access

**Published:** 1897-04-24

**Authors:** 


### Medical Progress and Hospital Clinics.

[*The Editor will be glad to receive offers of co-operation and contributions from members of the profession. All letters should be addressed to* The Editor, at the Office, 28 & 29, Southampton Street, Strand, London, W.C.]

### MALARIA AND ITS MODE OF ACCESS.

In view of the greatly increased exactitude of the conceptions regarding the nature of malarial diseases to which we have recently attained, it becomes a matter of great importance that entirely fresh clinical and statistical observations should be made as to the manner in which the infection enters the human body. The old idea that malaria was a mere matter of earth and air, a miasma rising from the ground and inhaled by the lungs, in favour of which such an immense amount of evidence can be brought forward by those who have lived in malarial countries, has for many years past been gradually giving way to the broader view that, at least as an alternative route, the infection can be carried by water; and, finally, the observations of Dr. Manson in regard to the life history of the malaria parasite outside the body, and the emphasis which he has laid on the part played by the mosquito in carrying the parasite back again from man to water, have made people additionally ready to accept the water-carriage of malaria as a matter to be accepted without question. It must, however, be stated that, however exact may be the knowledge we possess about this curious parasite, so far as it goes, and so far as it concerns that phase of its existence which it passes in man's body, we are in very great ignorance as to its proceedings when man is not present. If we are to believe that the fitness of any living thing for its surroundings is always the outcome of a long process of evolution it is impossible, in view of the perfect adaptation of the organism of malaria for its position as a parasite of the blood corpuscle, to believe for a moment that its occurrence there is a merely accidental thing, and we are driven to the conclusion that in the absence of man, and long before man appears on the scene at all, these organisms must have been parasites of the corpuscles of some creature or other, but what that creature is we do not know.

It is clear enough that the parasite passes from man to the mosquito, as, indeed, it might to any creature that sucked his blood, but it is by no means clear how the parasite gets back to man, or, indeed, that the parasites swallowed by the mosquito, or even their descendants, ever get back to man at all, and until we know more on this subject it is, we think, premature to accept the facts observed regarding the infection of the mosquito as in any way proving the water carriage of the disease to man.

Our knowledge of the parasitic nature or of the disease has, however, greatly cleared the ground in another direction. There can be no doubt that in malarial countries the term malaria has been made to cover a multitude of diseases, and that a great many cases hitherto looked upon as malarial have been really due to infection by the typhoid organism or by some analogous and as yet undifferentiated form of microbe. By examination of the blood these cases can now be sorted out, and it is becoming increasingly clear that many of the cases which crowd the literature of the subject, and have been looked upon as giving proof of the water carriage of malaria, must be received with grave suspicion so far as their malarial nature is concerned.

What we want, then, is a re-investigation of the whole question by direct observation. Knowing the nature of the infective organism, we want to know by what means it enters man's body. The matter is one of the greatest importance to us in our "little wars" in tropical countries. That troops and explorers in these regions must always drink purified water if they would keep free from various tropical diseases is clear, but until we know more about the mode by which the malarial poison reaches man than we do at present, it will not do to allow it to be thought that even the purest of water supplies is any sense prophylactic against malaria, or gives any excuse for neglecting the old and well-proved methods of obtaining protection from that disease.

